# Human Vocal Attractiveness as Signaled by Body Size Projection

**DOI:** 10.1371/journal.pone.0062397

**Published:** 2013-04-24

**Authors:** Yi Xu, Albert Lee, Wing-Li Wu, Xuan Liu, Peter Birkholz

**Affiliations:** 1 Department of Speech, Hearing and Phonetic Sciences, Division of Psychology and Language Sciences, University College London, London, United Kingdom; 2 Clinic of Phoniatrics, Pedaudiology, and Communication Disorders, University Hospital Aachen and RWTH Aachen University, Aachen, Germany; Baycrest Hospital, Canada

## Abstract

Voice, as a secondary sexual characteristic, is known to affect the perceived attractiveness of human individuals. But the underlying mechanism of vocal attractiveness has remained unclear. Here, we presented human listeners with acoustically altered natural sentences and fully synthetic sentences with systematically manipulated pitch, formants and voice quality based on a principle of body size projection reported for animal calls and emotional human vocal expressions. The results show that male listeners preferred a female voice that signals a small body size, with relatively high pitch, wide formant dispersion and breathy voice, while female listeners preferred a male voice that signals a large body size with low pitch and narrow formant dispersion. Interestingly, however, male vocal attractiveness was also enhanced by breathiness, which presumably softened the aggressiveness associated with a large body size. These results, together with the additional finding that the same vocal dimensions also affect emotion judgment, indicate that humans still employ a vocal interaction strategy used in animal calls despite the development of complex language.

## Introduction

Physically attractive men and women enjoy enhanced success in dating, job applications and elections [1]–[3], and they receive more support during social interactions [4]. Attractiveness is closely related to physical properties like facial features, body shape and other secondary sexual characteristics [1]–[3], [5]. Voice, as one of the secondary sexual characteristics, can also affect perceived attractiveness of an individual [6], [7]. As found by Zuckerman and Driver [6], an attractive voice can also help the judgment of facial attractiveness. Several acoustic cues have been identified to be associated with voice attractiveness. Male voices with lower fundamental frequency are in general preferred by female listeners [5], [8], [9]. Female voices with higher fundamental frequency and higher formant frequencies are heard as more attractive by male listeners [10]. Women raise their voice pitch when speaking to men they find attractive [11].

What is not clear is why specific characteristics are associated with an attractive voice. One possibility is that an attractive voice is closer to the averaged voice [12], thus is analogous to an averaged face, which is known to have increased facial attractiveness [13]. Another possibility is that a voice is attractive when it signals desirable attributes in a potential mate, e.g., masculinity, social dominance and health of men [5], [14], [15], or youth, reproductive health and mate quality of women [16], [17].

Further insight could be gained by considering the dimorphism of male and female vocal anatomies. The male vocal tract is longer than the female vocal tract, which leads to closer distances between the formants of vowels [18], [19]. Male vocal folds are longer than those of females, leading to a lower fundamental frequency [20]. On the other hand, the female voice often has a breathier quality than the male voice [21], [22] due to an incomplete closure of the vocal folds [23], [24]. The male-female vocal dimorphism could be explained by Morton’s theory of animal behavior [25], according to which many birds and mammal species use vocal characteristics that indicate body size to signal their intentions:

- Harsh, relatively-low frequency sounds indicate that the sender is likely to attack if further approached or the receiver stays in the same distance.
- More pure tone like, high frequency sounds indicate that the sender is submissive or appeasing if approached or if approaching, or fearful.

Here pattern A is to project a large body size so as to threaten the receiver, because a larger animal has a better chance at winning a physical confrontation. Pattern B is to project a small body size to attract the receiver, because a smaller animal is less likely to be a threat. A projected small body size also has an added benefit of mimicking an infant so as to elicit parental care [25].

Following this theory, the longer vocal folds of human males may have evolved under a selection pressure to compete with other males in achieving dominance for the sake of gaining access to female mates [26]. Likewise, the longer vocal tract of males may have evolved under the same pressure, as it may also reflect a larger body size [26]. Extending the mechanism further, the shorter vocal folds and vocal tract of females may have developed under a pressure in the opposite direction, i.e., to project a small body size in order to attract male mates. A similar pressure may have led to the development of the smile, which signals sociability by shortening the effective length of the vocal tract [26]. This proposal has been supported by the finding that speech sounds synthesized with shorter vocal tract and higher pitch is heard as both from a smaller person and happier, while sounds synthesized with longer vocal tract and lower pitch are heard as both from a larger person and more angry [27], [28].

Furthermore, a vocalization that projects a small body size should also be more pure-tone like according to Morton [25]. Normal speech cannot directly resemble pure tones, however, because the harmonics of the complex speech sounds carry essential phonetic information [18], [19]. But the next closest would be a breathy voice quality. A breathy voice is produced with an incompletely closed glottis, which results in glottal waveforms that are relatively round, i.e., lacking a complete cessation of glottal airflow [19]. The spectra of such relatively round waveforms are more tilted, having reduced higher frequency energy and relatively prominent first harmonic, i.e., the harmonic corresponding to the fundamental frequency [24]. Compared to a modal voice, i.e., one with complete glottal closure, a breathy voice is therefore more pure-tone like and thus probably more “pleasant” auditorily. Breathy voice is known to be more prevalent among females than among males [21], [22], [29]. Thus it is likely that breathiness may also contribute to female vocal attractiveness. In contrast, a pressed voice, with the opposite spectral quality as breathy voice, could potentially decrease attractiveness.

While recent research has shown additional factors that influence perceived attractiveness, e.g. menstrual cycle and self-perceived health [30], [31], a systematic explanation for the correlation between certain acoustical parameters and an attractive voice per se is not yet in place. In the case of male voice, not much is known about its attractiveness other than the importance of being low-pitched [5], [8]. Here we used perception experiments to test whether manipulation of acoustic parameters along the body-size projection dimensions can effectively change the attractiveness of full utterances to the opposite sex. The sentences used were in English, either humanly spoken or purely synthetic with different voice qualities, and then acoustically manipulated in terms of fundamental frequency (F_0_) height, F_0_ slope, F_0_ range and formant dispersion (distribution of formants along the frequency dimension). We also tested whether the same vocal properties affect the perception of vocal emotion, so as to establish a link between vocal attractiveness and vocal expression of emotions.

## Methods and Results

**Figure 1:**
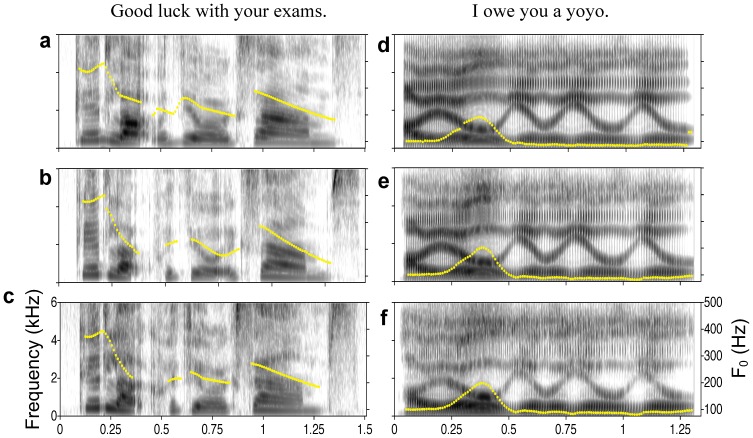
The base setences. Spectrograms and pitch tracks (dotted yellow lines) of the base sentences in Experiment 1 (a–c) and Experiments 2–5 (d–f). In order, the three rows of graphs represent utterances in normal, breathy, and pressed voices.

In the first experiment, 10 young male native speakers of English (average age: 23) heard a female voice saying the sentence “Good luck with your exams” in Standard Southern British English, and judged the attractiveness of the voice on a 5-level scale, with 5 being the most attractive. The stimulus sentences were pre-recorded by a female speaker in three voice qualities–normal, breathy and pressed, without any emotional involvement (Figure 1a–c). The sentences were then digitally modified in terms of median pitch, formant dispersion and sentence-final pitch slope, see Table 1, along the directions of signaling a small body size and happiness, or large body size and anger [27], [28]. The specific amounts of these modifications were based on previous studies on emotion [27], [28], [32], pilot testing, and specifications of the VocalTractLab software [33]. Further methodological details can be found in the Methodology section.

**Table 1:** Parameters and their changes applied to the base sentences for the preparation of the stimuli.

Body size projection	Voice quality	Formant shift ratio	Pitch shift	Final F_0_ slope (Exp. 1)	Pitch range – Ratio to base (Exp. 2–5)
Small	Breathy	1.1	+2 st	+15 st/s	2.0
↓	Neutral	1	0	1	1
Large	Pressed	0.9	−2 st	−15 st/s	0.25

A formant shift ratio greater than 1 increases the frequency of all formants (A ratio of 1.1 simulates a shortening of the vocal tract by approximately 10%, and a ratio of 0.9 a lengthening by 10%.). Pitch shift modifies the median pitch of an entire sound. Final F_0_ slope modifies the pitch slope of the final syllable in “exam” in Experiment 1. Pitch range expands or compresses the dynamic F_0_ range of the entire sentence. The columns are independent of each other.

**Figure 2:**
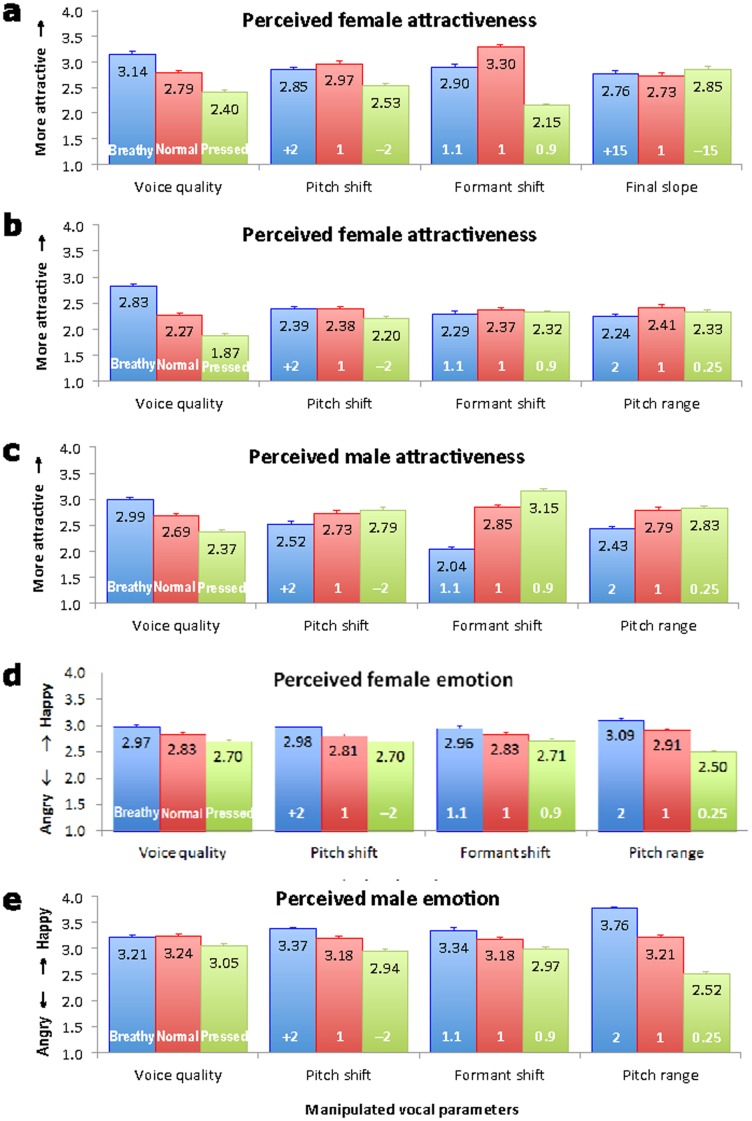
Judgment ratings. Judgments of voice attractiveness (a–c) and emotion (d–e), on a scale of 1–5, as a function of Voice quality, Pitch shift, Formant shift, Final F_0_ slope and Pitch range. Each row of the graphs (a–e) corresponds to Experiments 1–5 respectively. In each bar, the black figures represent mean rating score, while parameter values are in white. The error bars are standard errors.

The judgments were in the expected directions, as shown in Figure 2a. Attractiveness is monotonically increased as voice quality goes from pressed to normal to breathy (F_2,18_ = 73.71, *p<*0.0001). Upward pitch shift increased attractiveness, (F_2,18_ = 11.00, *p* = 0.0008), but the difference between the normal and raised pitch was not significant (Bonferroni/Dunn post-hoc), indicating that the pitch of the female speaker was sufficiently high in terms of attractiveness, but lowering it made the voice less attractive. Upward formant shifts also increased attractiveness overall (F_2,18_ = 21.31, *p<*0.0001), but the difference between the normal (ratio = 1.0) and the raised (ratio = 1.1) was not significant, indicating a lack of further benefit when the vocal tract was shortened beyond that of the original female speaker. There is no effect of final F_0_ slope, suggesting that this particular linguistic factor is not directly related to attractiveness.

These results appear to be consistent with the size-projection hypotheses. In terms of pitch and formant dispersion, the original values, which may resemble the population mean, are apparently sufficiently attractive, which also agrees with the averageness theory [12]. But only deviations toward a larger body size (lowered pitch and increased formant dispersion) reduced attractiveness, which agrees better with the size-projection hypothesis. Furthermore, increasing breathiness monotonically increased attractiveness, as shown in Figure 2a.

**Figure 3:**
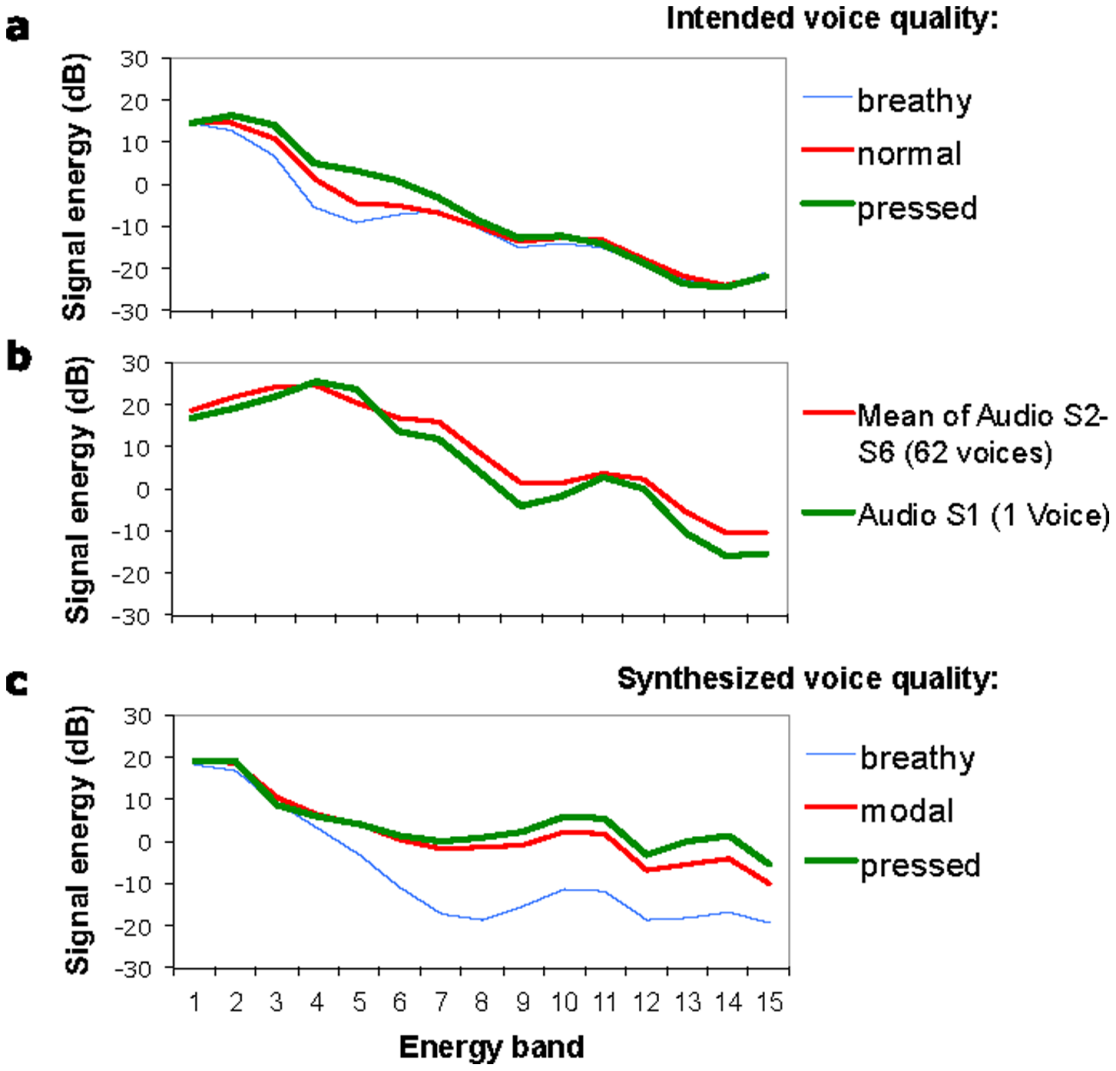
Band energy profiles. Band energy profiles of speech sounds. Each profile consists of fifteen signal energy values computed from overlapping spectral bands of 500-Hz bandwidth: 0–500, 250–750, 500–1000, … 3250–3750, 3500–4000. a, Mean band energy profiles of all 6 vowels in the three base sentences of Experiment 1, each with an intended voice quality. b, Band energy profiles of two sample files from Bruckert et al. (2010). c, Profiles of three synthetic sentences used in Experiment 2–5, each with a synthetic voice quality.

To make sure that the voice quality types were effectively produced by our speaker as intended, we performed a number of acoustic analyses. The first is an energy-band analysis of the vowel spectra, shown in Figure 3a, using a method found to be effective in detecting subtle voice quality differences from continuous speech [34]. The analysis produces energy profiles each consisting of signal energy values of fifteen overlapping spectral bands of 500-Hz bandwidth (see Methodology appendix for more details). These energy band profiles show that as voice goes from pressed to breathy, more spectral energy is concentrated toward the lower frequency. In addition, we took a number of measurements commonly used to characterize voice quality, as shown in the upper part of Table 2. As the intended voice goes from breathy to pressed, H1–H2*, H1–A1* and H1–A3* all show decreasing values (except H1–A3* of pressed voice), indicating an overall reduction of spectral tilt. Also the center of spectral gravity moves upward in frequency across the three intended voice types, again indicating decreased spectral tilt. Thus, with only a single exception, all the measurements indicate that the speaker produced breathy, normal and pressed voice qualities as intended.

**Table 2:** Measurements of voice quality.

Measurement Speech type	Intended voice	H1–H2*	H1–A1*	H1–A3*	Center of Spectral Gravity
	breathy	2.08	4.22	33.10	456.9
Natural	normal	–0.60	–0.22	32.00	537.3
	pressed	–1.63	–1.25	33.75	659.6
	breathy	–0.16	–2.09	28.63	377.6
Synthetic	normal	–0.65	–4.98	14.13	535.8
	pressed	–1.28	–7.91	6.76	656.3

The first three measurements are in dB; the last measurement is in Hz.

In Experiment 1, the sentences with different voice qualities had to be spoken by the human speaker in separate utterances. As a result, the sentences differed not only in voice quality as intended, but also in other prosodic dimensions, as can be seen in Figure 1a–c, and so possible confounds could not be fully ruled out. In Experiments 2–5 we thus used entirely artificial speech as stimuli where we had full control over all prosodic parameters. The base stimuli for these experiments were created using VocalTractLab – an articulatory speech synthesizer [33], [35] which allows us to synthesize arbitrary utterances based on a specification of the constituting elementary speech movements (gestures) in high-quality. As found in a recent study, the new two mass model of the vocal folds in VocalTractLab could generate voice qualities that were convincingly heard by listeners as breathy, normal and pressed, at a perceptual level much higher than the classical two mass model [33]. We created the sentence “I OWE you a yoyo” with an emphasis on the word “owe” (Figure 1d–f), which was manually modeled after an utterance by a male speaker of Southern British English. Three synthetic versions of the sentence were generated, each in one of three voice qualities–pressed, modal and breathy, while other parameters were kept identical. As shown in Figure 3c, an energy-band analysis of the three versions of the sentence shows similar profile separation as in the natural sentences in Experiment 1, with center of gravity values of 585 Hz for the pressed voice, 473 Hz for the modal voice and 353 Hz for the breathy voice, as shown in the lower right part of Table 2. Also shown in lower rows of Table 2 are values of H1–H2*, H1–A1* and H1–A3*. All these measurements indicate decreased spectral tilt as the intended voice goes from breathy to pressed (see Sound S9, S10, S11 for the base sentences with the three intended voice qualities). The female versions of the sentences were synthesized by increasing F_0_ median by 12 semitones (1 octave) and Formant Shift by 0.2, while other things remained equal.

We then used the same method as in Experiment 1 to manipulate Formant shift, Pitch shift, and Pitch range (see Supporting Information for audio samples and the Praat script Script S3 that performs the acoustic manipulation), see Table 1 (column 6). Pitch range was tested instead of intonation slope as in Experiment 1 because it has been found to be relevant for the perceptual rating of friendliness and happiness [32]. Listeners (N = 32, 16 female) were played the stimuli of the opposite sex, and asked to judge the attractiveness of the speaker. The ratings of female vocal attractiveness (Experiment 2), as shown in Figure 2b, were in line with those of Experiment 1. Increased breathiness again monotonically improved attractiveness (F_1.13,16.98_ = 40.153, *p<*0.001). A post-hoc Bonferroni test confirmed that breathy voice was significantly more attractive than modal voice and pressed voice (*p*<0.001). Lowered pitch was heard as less attractive (F_1.12,16.80_ = 3.793, *p = *0.065), though the main effect of pitch height was only marginally significant. The main effect of Formant shift (or any of the post-hoc effects), unlike in Experiment 1, was not significant, but on the whole the original Formant value was perceived as the most attractive. The new parameter, Pitch range, did not show a significant main effect either, but Figure 2b showed that normal and narrow pitch ranges were generally perceived as more attractive. It thus can be concluded that a female voice sounds attractive when it is breathy, moderately high-pitched, and with moderately dispersed formants and normal or narrow pitch ranges (see audio samples in Supporting Information).

For male voice, there is a paradox for the size-projection hypotheses. If an attractive male voice is the direct opposite of an attractive female voice, it would have low pitch, densely distributed formants and pressed voice quality. But these attributes have been proposed to signal aggressiveness, because the large body size they project would help an animal or human individual to prevail in a confrontation [25], [26]. And they have been shown to signal anger to human listeners, thus linking anger to aggressiveness [32]. Would it really be the case that an attractive male voice is an aggressive and angry one? Or is there at least one attribute that is used to soften the aggressiveness? This puzzle was further studied in Experiment 3, in which we used the same base sentences as in Experiment 2 except that the overall pitch and formant dispersion values were adjusted to be male-appropriate. Then the same parameter modifications as in Experiment 2 were applied to generate the perceptual stimuli. Sixteen female listeners judged the attractiveness of these stimuli. As can be seen in Figure 2c, attractiveness of male voice was increased by downward formant shift (F_2,30_ = 66.788, *p<*0.001) and downward pitch shift (F_2,30_ = 14.493, *p<*0.001), both of which are consistent with anger and aggressiveness. However, attractiveness monotonically increased with breathiness (F_1.21,18.19_ = 8.221, *p = *0.007) (Figure 2c), just as it did with the female voice. Also like with the female voice, normal and reduced pitch ranges sounded more attractive (F_1.16,17.42_ = 11.039, *p = *0.003). Thus to a female listener, an attractive male voice is one that projects a large body size with lowered pitch and densely distributed formants. However, like its female counterpart, an attractive male voice is also breathy and with normal or narrow pitch range (see audio samples in Supporting Information).

To further establish a link between vocal attractiveness and emotion, Experiments 4 and 5 examined how vocal anger vs. happiness were perceived by the opposite sex. The same procedures and stimuli were used as in Experiment 2, except that this time listeners (N = 32, 16 female) were to give ratings along a 5-level Angry–Happy scale. The rating of female vocal emotion by male listeners (Experiment 4), shown in Figure 2d, partly resembled those of female attractiveness (Figure 2a, 2b). The main effect of voice quality was marginally significant (F_2,30_ = 3.297, *p = *0.051), a breathy female voice sounded happier, while pressed voice was always perceived as angrier. Happiness was also associated with greater formant dispersion (F_2,30_ = 7.468, *p = *0.002) and higher pitch (F_2,30_ = 6.997, *p = *0.003), although only raised pitch was significantly happier than the original (*p* = 0.004) and lowered pitch (*p = *0.03), according to Post-hoc Bonferroni test. Unlike for attractiveness, however, it was the expanded pitch range that was perceived as happier (F_2,30_ = 8.648, *p = *0.001). Experiment 5 showed that anger versus happiness in male voice shared similar parameters as in female voice. An angry voice also had more densely distributed formants (F_1.34,20.11_ = 11.422, *p = *0.001), which signals a large body size. Also like in Experiment 4, the happiness of a male voice increased with pitch range (F_1.13,16.97_ = 54.529, *p<*0.001), and the ratings of the 3 ranges were significantly different from one another in a post-hoc Bonferroni test (*p<*0.01). Likewise, a happy voice is higher-pitched (F_1.15,17.21_ = 27.542, *p<*0.001), with the ratings of the 3 pitch heights being significantly different from one another. However, the main effect of voice quality was non-significant (see Supporting Information for auditory samples of synthetic happy and angry voice).

## Discussion

The results presented here show that female voices rated as more attractive were breathy, high pitched (though not too high), with widely dispersed formants (again, not too dispersed), and all these qualities are consistent with the projection of a relatively small body size. In contrast, male voices rated as more attractive were low-pitched with densely distributed formants, both of which project a large body size. But male voice attractiveness also increased with breathiness, which projects a small body size. These results are largely consistent with the hypothesis that vocal attractiveness is achieved with the size projection mechanism also used in animal calls [27], [28], [36], [37]. But the breathiness in the male voice attractiveness rating is intriguing, as it could be a way of neutralizing the aggressiveness associated with a large body size [25].

These results, when taken together with the dimorphism between female and male vocal anatomy, suggest that what makes the voice attractive are mostly properties that enhance the characteristics already in the averaged voice of the sex: high pitch, dispersed formants and breathiness in female voice, and low pitch and long vocal tract in the male voice. These findings may therefore explain why averaged voices are more attractive than certain individual voices [12]. That is, the continued reproductive success of the human species means that the average individual attributes, including those of the voice, must have been sufficiently attractive to the opposite sex. But for any individual to stand a better-than-average chance, it would be desirable to exaggerate the characteristics that further enhance attractiveness. And the enhancement, based on the present findings, seems to be based on the principle of body size projection in the case of voice.

The present results also show, for the first time, a clear effect of voice quality on vocal attractiveness. In fact, voice quality is by far the most important attribute, because a breathy voice, whether female or male, was always heard as the most attractive. Also, the fact that for female voice there seems to be a limit to the attractiveness-enhancing effects of raising pitch and dispersing formants (Figure 2a–b) (presumably because they have made the voice too child-like), may explain why breathiness is more important for female than male voice attractiveness [21], [38], and why breathy voice is the most relevant quality for male-to-female transsexuals [38], [39], and probably even why the posterior glottal opening, which leads to a breathy voice, is more consistent in young women than in both young men [23] and elderly women [40]. The importance of breathiness in increasing the attractiveness of female as well as male voice has clear practical implications for areas like speech-based technology, speech and voice counseling, voice surgery and voice therapy for transsexuals.

Finally, although it is widely accepted that humans are genetically related to other animal species, direct scientific evidence that the human speech also shares similarities with information systems in nonhuman species is rare. The findings of the present study indicate that, despite the development of highly complex language capable of conveying fine subtleties in meaning, humans still use an encoding strategy similar to the one widely used by nonhuman animals for guaranteeing success in survival and reproduction.

The present study is not without limitations. The acoustic manipulation of human voice could have somehow reduced its naturalness, although there were no such complaints from the listening subjects. The voice qualities generated by the articulatory synthesizer, though better than any other we have heard before, still has room for improvement. Future studies can investigate the perception of vocal attractiveness by listeners of the same gender, or examine whether listeners from different linguistic and cultural backgrounds have differential preference for an attractive voice.

### Conclusion

The present study has shown evidence that human vocal attractiveness is encoded along the same size projection dimension that has been suggested for encoding animal calls and human emotional expressions [25], [27], [28], [32]. That is, a female voice sounded attractive when it was breathy, moderately high-pitched, and with moderately dispersed formants, all of which signal a relatively small body size. A male voice sounded attractive when it was low pitched and with densely distributed formants, both of which signal a large body size. But a male voice also sounded attractive when it was breathy, which presumably reduced the aggressiveness associated with the large body size projected by the low pitch and densely distributed formants. In general, therefore, the current findings demonstrate the potential of the evolutionarily-based approach [25], [26] to link areas of research that have been so far quite separated, such as emotion, personal attributes, sexual behavior and dimorphism, and social interactions.

## Methodology

### Experiment 1

The sentence, “Good luck with your exams,” was spoken by a female speaker of South-Eastern British English, aged 23 years, in three voice qualities: normal, breathy and pressed, with no emotional or attitudinal involvement. The three base sentences were then normalized in intensity and pitch contours with the Praat program [41]. Pitch contours were normalized by using an intonation modeling program [42] to extract the synthesis parameters from the normal-voice sentence and then apply them to all three sentences. Also using the synthesis program the F_0_ slope of the final syllable in the word “exam” was modified into normal, steep and shallow. The actual stimuli were then generated by modifying the base sentences in terms of Formant shift and Pitch shift, using a custom-written script that applied the “Change gender” function in the Praat program (see Script S2 in Supporting Information for the Praat script that performs the acoustic manipulation). Ten young male native speakers of English participated as listening subjects. They listened to the stimulus sentences through headphones in a quiet room, and judged the attractiveness of each sentence on a five-level scale.

### Experiment 2–5

The base sentence, “I owe you a yoyo”, was created with VocalTractLab 2.0–a digital articulatory speech synthesizer [33], [35]. The sentence was modeled manually after an utterance spoken by a male speaker of Southern British English. Three synthetic versions of the sentence were generated by VocalTractLab, each in one of three voice qualities–pressed, modal and breathy, while other parameters were kept identical. The base sentences were then modified with a Praat script (see Script S3 in Supporting Information for the Praat script that performs the acoustic manipulation). Sixteen young males and sixteen young females participated as subjects. They listened to the stimulus sentences through headphones in a quiet room, and judged the attractiveness and emotion of each sentence on a five-level scale.

### Stimuli

#### Experiment 1

The sentences were recorded in a quiet room with a head-mounted condenser microphone (Countryman Isomax hypercardiod). To check if the speaker inadvertently varied vowel formants with the voice quality, we measured the frequencies of the first three formants of all six vowels in each sentence and calculated formant dispersion (averaged distance between adjacent formants) with formula (1) [43].
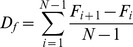
(1)


The mean formant dispersions were 1182, 1195 and 1138 Hz for breathy, normal and pressed voice, respectively, but the differences were not statistically significant (*p* = 0.279).

The three base sentences were then normalized in intensity and pitch contours. Intensity was normalized by equalizing the maximum amplitude of all the sentences with the Scale peak command in the Praat program [41]. Pitch contours were normalized by using an intonation modelling program [42] to extract the synthesis parameters from the normal-voice sentence and then apply them to all the three sentences. Also using the synthesis program the F_0_ slope of the final syllable in the word “exam” was modified into normal, steep and shallow. The speech rates of the three sentences were similar (4.16–4.23 syllables/second) and were not further normalized to avoid possible phonetic distortion.

The actual stimuli were then generated by modifying the base sentences in terms of Formant shift and Pitch shift, using a custom-written script that applied the “Change gender” function in the Praat program (see Supporting Information for the Praat script). In total, 81 stimuli were prepared (3 voice qualities × 3 formant shift ratios × 3 pitch shifts × 3 final F_0_ slopes).

#### Experiment 2–5

The base sentence, “I owe you a yoyo”, was created with VocalTractLab 2.0 – a digital articulatory speech synthesizer [33], [35]. The sentence was modeled manually after an utterance spoken by a male speaker of Southern British English. Three synthetic versions of the sentence were generated, each in one of three voice qualities–pressed, modal and breathy, while other parameters were kept identical. The voice quality manipulation was done by a modified two-mass model implemented in VocalTractLab 2.0 [33]. The breathy, normal and pressed voice were created by setting the parameter “upper-lower rest displacement” of the vocal fold model at 0.30 mm, 0.10 mm and −0.10 mm, respectively. The synthetic sentences were then modified with a script that applied the “Change gender” function in the Praat program [41] (see Supporting Information for the Praat script). In total, 81 stimuli were prepared (3 formant shift ratios × 3 pitch shifts × 3 pitch ranges × 3 voice qualities).

### Voice Quality Analysis

All the voice quality analyses were performed with a Praat script (see Supporting Information).

The band energy analysis was adopted from the EQ15 analysis in Surendran (2008) [34]. It has fifteen overlapping bands of 500 Hz bandwidth between 0 and 4000 Hz : 0–500, 250–750, 500–1000,…, 3250–3750, 3500–4000. The energy of each band is measured in dB using Praat’s Get power function.

H1–H2*, H1–A1* and H1–A3* were approximates of the previously proposed measurements H1–H2, H1–A1 and H1–A3 [29], where H1 and H2 refer to the amplitudes of the first and second harmonics of a vowel, and A1 and A3 refer to the amplitude of the first and third formants. Our approximations of these measurements are based on the power differentials taken at the median pitch of a vowel, its double frequency (H2), average of the 2^nd^ and 3^rd^ energy bands (A1) and average of the 11^th^, 12^th^ and 13^th^ energy bands (A3). See Script S1 in Supporting Information for the algorithms.

### Listening Tests

#### Experiment 1

Ten young males with an average age of 23 years participated as subjects. They were native speakers of English with no self-reported speech or hearing impairments. They listened to the stimulus sentences, played in randomised order, through Sennheiser HD 265 linear headphones in a quiet room, and judged the attractiveness of each sentence. They could listen to each stimulus up to three times, although in most cases they listened to each stimulus only once. All participants were paid a small remuneration for their time.

#### Experiment 2–5

Sixteen males (age: 19–48, mean age = 25.8) and sixteen females (age: 18–30, mean age = 22.5) participated as subjects. They were native speakers of English with no self-reported speech or hearing impairments. No subjects in these experiments took part also in Experiment 1. They listened to the stimulus sentences, played in randomised order, through Sennheiser HD 265 linear headphones in a quiet room, and judged the attractiveness and emotion of each sentence, in separate sessions. In each experiment, listeners first attempted a practice trial where they rated 12 utterances; subsequently 243 responses were collected from every listener for analysis. They could listen to each stimulus up to three times, although in most cases they listened to each stimulus only once. There was an optional break after every 81 utterances. In all four experiments, participants were paid a small remuneration for their time.

### Analysis of Listening Results

Results of the attractiveness and emotion ratings (Experiments 1–5) were extracted from Praat for statistical analyses. We performed analysis of variance (ANOVA) on data of each of the 5 experiments, with the fixed factors voice quality, formant dispersion, and pitch height. There was a further factor analysed, namely final slope for Experiment 1 and pitch range for Experiments 2–5. Significant main effects were subsequently verified using post-hoc Bonferroni test. These results were used then to generate the graphical illustrations in Figure 2.

#### Ethics statement

Appropriate procedures were followed in obtaining written informed consent from the subjects of all experiments above. This study has been approved by the UCL Research Ethics Committee (SHaPSetXU002).

## Supporting Information

Script S1 **Praat script for computing band energy and centre of gravity.**
(PDF)

Script S2 **Praat script for generating stimuli for Exp. 1.**
(PDF)

Script S3 **Praat script for generating stimuli for Exp. 2–5.**
(PDF)

Sound S1 **An example of most attractive synthetic female voice.** This audio was created with Praat parameters formant_shift_ratio = 1.0, pitch shift = 0, pitch range = 0.25, and VocalTractLab parameter upper-lower rest displacement = 0.30 mm.
(WAV)

Sound S2 **An example of least attractive synthetic female voice.** This audio was created with Praat parameters formant_shift_ratio = 1.1, pitch shift = −2, pitch range = 0.25, and VocalTractLab parameter upper-lower rest displacement = −0.10 mm.
(WAV)

Sound S3 **An example of most attractive (based on least dimensional scores) synthetic male voice.** This audio was created with Praat parameters formant_shift_ratio = 0.9, pitch shift = −2, pitch range = 0.25, and VocalTractLab parameter upper-lower rest displacement = 0.30 mm.
(WAV)

Sound S4 **An example of least attractive synthetic male voice.** This audio was created with Praat parameters formant_shift_ratio = 1.1, pitch shift = 2, pitch range = 2.0, and VocalTractLab parameter upper-lower rest displacement = −0.10 mm.
(WAV)

Sound S5 **An example of most happy synthetic female voice.** This audio was created with Praat parameters formant_shift_ratio = 1.1, pitch shift = 2, pitch range = 2.0, and VocalTractLab parameter upper-lower rest displacement = 0.30 mm.
(WAV)

Sound S6 **An example of most angry synthetic female voice.** This audio was created with Praat parameters formant_shift_ratio = 0.9, pitch shift = −2, pitch range = 0.25, and VocalTractLab parameter upper-lower rest displacement = −0.10 mm.
(WAV)

Sound S7 **An example of most happy synthetic male voice.** This audio was created with Praat parameters formant_shift_ratio = 1.1, pitch shift = 2, pitch range = 2.0, and VocalTractLab parameter upper-lower rest displacement = 0.30 mm.
(WAV)

Sound S8 **An example of most angry synthetic male voice.** This audio was created with Praat parameters formant_shift_ratio = 0.9, pitch shift = −2, pitch range = 0.25, and VocalTractLab parameter upper-lower rest displacement = −0.10 mm.
(WAV)

Sound S9 **The synthetic base sentence in modal voice.** This audio was created with VocalTractLab, with parameter upper-lower rest displacement = 0.10 mm.
(WAV)

Sound S10 **The synthetic base sentence in breathy voice.** This audio was created with VocalTractLab, with parameter upper-lower rest displacement = 0.30 mm.
(WAV)

Sound S11 **The synthetic base sentence in pressed voice.** This audio was created with VocalTractLab, with parameter upper-lower rest displacement = –0.10 mm.
(WAV)
