# Supplementary material for: Human Vocal Attractiveness as Signaled by Body Size Projection
Source: PLoS One. 2013 Apr 24;8(4):e62397. doi: 10.1371/journal.pone.0062397 (PMC3634748; doi:10.1371/journal.pone.0062397)
Supplement: Script S1 — Praat script for computing band energy and centre of gravity. (PDF) [file pone.0062397.s001.pdf]

```
# BID.praat
# Written by:   Yi Xu   2/12/13
form Start
    integer Ifile 1
    real step_size 250
    real band_size 500
endform

directory$ = "./"
Create Strings as file list... list 'directory$'*.wav
numberOfFiles = Get number of strings
if !numberOfFiles
    Create Strings as file list... list 'directory$'*.WAV
    numberOfFiles = Get number of strings
endif
if !numberOfFiles
    exit There are no sound files in the folder!
else
    Write to raw text file... 'directory$'FileList.txt
endif

hastitleline = 0
for ifile from ifile to numberOfFiles
    select Strings list
    fileName$ = Get string... ifile
    Read from file... 'directory$''fileName$'
    starttime = Get start time
    endtime = Get end time
    name$ = fileName$ - ".wav" - ".WAV"
    titleline$ = "Filename"
    energy_titleline$ = "Filename"
    energyline$ = name$
    call BID 'fileName$'
    if !hastitleline
        filedelete Energy.txt
        filedelete Center_of_gravity.txt
        filedelete Harmonics.txt
        fileappend Energy.txt 'energy_titleline$''newline$'
        fileappend Center_of_gravity.txt 'name$'    Center_of_gravity'newline$'
        fileappend Harmonics.txt Filename   H1-H2*  H1-A1*  H1-A3*'newline$'
        hastitleline = 1
    endif
    fileappend "Energy.txt" 'energyline$''newline$'
    fileappend "Harmonics.txt" 'name$'  'h1_H2' 'h1_A1' 'h1_A3''newline$'
    fileappend "Center_of_gravity.txt" 'name$'  'center_gravity''newline$'
    select Sound 'name$'
    Remove
endfor

procedure BID file_name$
    center = step_size
    select Sound 'name$'
    To Spectrum... yes
    center_gravity = Get centre of gravity... 2
    select Sound 'name$'
    To Pitch... 0.02 30 600
```

```
    median_pitch = Get quantile... starttime endtime 0.5 Hertz
    for band from 1 to 15
        if !hastitleline
            energy_titleline$ = "'energy_titleline$'    'center'"
        endif
        floor = center - 0.5*band_size
        ceiling = center + 0.5*band_size
        select Sound 'name$'
        Filter (pass Hann band)... floor ceiling 100
        energy = Get power... 0 0
        energy = 10 * log10(50000*energy)
        energy'band' = energy
        Remove
        energyline$ = "'energyline$'    'energy'"
        center += 0.5*band_size
    endfor

    center = median_pitch
    bandwidth = 2 * median_pitch
    for band from 1 to 2
        floor = center - 0.5*bandwidth
        ceiling = center + 0.5*bandwidth
        select Sound 'name$'
        Filter (pass Hann band)... floor ceiling 100
        energy = Get power... 0 0
        energy = 10 * log10(50000*energy)
        h'band' = energy
        Remove
        center += median_pitch
    endfor
    h1_H2 = h1 - h2
    h1_A1 = h1 - (energy2 + energy3) / 2
    h1_A3 = h1 - (energy11 + energy12 + energy13) / 3
endproc
```
