# Supplementary material for: Human Vocal Attractiveness as Signaled by Body Size Projection
Source: PLoS One. 2013 Apr 24;8(4):e62397. doi: 10.1371/journal.pone.0062397 (PMC3634748; doi:10.1371/journal.pone.0062397)
Supplement: Script S2 — Praat script for generating stimuli for Exp. 1. (PDF) [file pone.0062397.s002.pdf]

```
# Stimulus_generator_attractiveness1
# Version 4.0
# Last update: 22 July, 2010
# Written by:   Yi Xu (yi.xu@ucl.ac.uk)

stimulusDirectory$ = "Stimuli/"
sourceDirectory$ = "Source/"
Create Strings as file list... list 'sourceDirectory$'*.aiff
numberOfFiles = Get number of strings

formant_shift_ratio1 = 1.1
formant_shift_ratio2 = 1.0
formant_shift_ratio3 = 0.9

pitch_shift1 = 2
pitch_shift2 = 0
pitch_shift3 = -2

for current_file from 1 to numberOfFiles
    select Strings list
    fileName$ = Get string... current_file
    call manipulation 'fileName$'
endfor

procedure manipulation file_name$
    for formant from 1 to 3
        for pitch from 1 to 3
            Read from file... 'sourceDirectory$''file_name$'
            source$ = selected$ ("Sound")
            Change gender...  100 600  formant_shift_ratio'formant' 0 1 1
            Rename... intermediateSound

            To Pitch... 0 75 600
            oldPitchMedian = Get quantile... 0 0 0.5 Hertz
            Down to PitchTier

            npoints = Get number of points

            for point from 1 to npoints
                pitch1 = Get value at index... point
                time = Get time from index... point
                newPitch = pitch1 * 2^(pitch_shift'pitch'/12)
                Remove point... point
                Add point... time newPitch
            endfor


            select Sound intermediateSound
            To Manipulation... 0.01 75 600
            plus PitchTier intermediateSound
            Replace pitch tier
            select Manipulation intermediateSound
            Get resynthesis (overlap-add)
            Rename...  intermediateSound1
            Write to WAV file... 'stimulusDirectory$''source$''formant''pitch'.wav
            plus Sound intermediateSound
            plus Sound intermediateSound1
            plus Manipulation intermediateSound
            plus PitchTier intermediateSound
            plus Pitch intermediateSound
            plus Sound 'source$'
            Remove
        endfor
    endfor
endproc
```
