# Supplementary material for: Human Vocal Attractiveness as Signaled by Body Size Projection
Source: PLoS One. 2013 Apr 24;8(4):e62397. doi: 10.1371/journal.pone.0062397 (PMC3634748; doi:10.1371/journal.pone.0062397)
Supplement: Script S3 — Praat script for generating stimuli for Exp. 2–5. (PDF) [file pone.0062397.s003.pdf]

```
# Stimulus_generator_attractiveness2
# Version 4.0
# Last update: 22 July, 2010
# Written by:   Yi Xu (yi.xu@ucl.ac.uk)

stimulusDirectory$ = "Stimuli3/"
sourceDirectory$ = "Source3/"
Create Strings as file list... list 'sourceDirectory$'*.wav
numberOfFiles = Get number of strings

formant_shift_ratio1 = 1.1
formant_shift_ratio2 = 1.0
formant_shift_ratio3 = 0.9

pitch_shift1 = 2
pitch_shift2 = 0
pitch_shift3 = -2

pitch_range1 = 2.0
pitch_range2 = 1
pitch_range3 = 0.25

for current_file from 1 to numberOfFiles
    select Strings list
    fileName$ = Get string... current_file
    call manipulation 'fileName$'
endfor

procedure manipulation file_name$
    for formant from 1 to 3
        for pitch from 1 to 3
            for range from 1 to 3
                Read from file... 'sourceDirectory$''file_name$'
                source$ = selected$ ("Sound")
                Change gender...  75 600  formant_shift_ratio'formant' 0 1 1
                Rename... intermediateSound

                To Pitch... 0 75 600
                oldPitchMedian = Get quantile... 0 0 0.5 Hertz
                newPitchMedian = oldPitchMedian * 2^(pitch_shift'pitch'/12)
                Down to PitchTier

                npoints = Get number of points

                for point from 1 to npoints
                    pitch1 = Get value at index... point
                    time = Get time from index... point
                    newPitch = pitch1 * 2^(pitch_shift'pitch'/12)
                    finalPitch = newPitchMedian + (newPitch - newPitchMedian) * pitch_range'range'
                    Remove point... point
                    Add point... time finalPitch
                endfor


                select Sound intermediateSound
                To Manipulation... 0.01 75 600
                plus PitchTier intermediateSound
                Replace pitch tier
                select Manipulation intermediateSound
                Get resynthesis (overlap-add)
                Scale peak... 0.99
                Rename...  intermediateSound1
                Write to WAV file... 'stimulusDirectory$''source$''formant''pitch''range'.wav
```

```
                plus Sound intermediateSound
                plus Sound intermediateSound1
                plus Manipulation intermediateSound
                plus PitchTier intermediateSound
                plus Pitch intermediateSound
                plus Sound 'source$'
                Remove
            endfor
        endfor
    endfor
endproc
```
